# A Case of Four Children at a Birth, Complicated with Hemorrhage

**Published:** 1847-12

**Authors:** John H. Donnell

**Affiliations:** Student of Medicine in the University of Louisville; Franklin, Illinois


Art. III.—*A Case of Four Children at a Birth, Complicated with Hemorrhage.* By Mr. John H. Donnell, Student of Medicine in the University of Louisville.

On the 6th of February last, I was called to visit Mrs. D., whom I found laboring under an attack of pneumonia. The disease yielded to active treatment, convalescence being established in the course of five or six days. On my first visit I was informed that the patient was six months advanced in utero-gestation; from her unusual size, I was led to suppose that her confinement would probably take place in less than a fortnight.

After she recovered, I heard nothing from her until the 31st of May, when I was called to visit her. On my arrival, I learned that one child was already born—a foot presentation; but on examining the patient, I found that there was another, and that the right shoulder was the presenting part. I ruptured the membranes, turned, and delivered by the feet. Another pain ensued in a short time, and a third child was delivered—a vertex presentation. At this period an alarming hemorrhage took place, for which I administered a large dose of tincture of ergot, and, being compelled to consult the safety of the mother, tied and divided the cords. Fortunately for my patient, the hemorrhage ceased very soon, and by the time it had done so I was satisfied of there being still another child, and that the vertex was presenting. I ruptured the membranes, expecting that the child would be speedily delivered; but after waiting for a pain and making a second examination per vaginam, I found the right hand had descended before the head. This I immediately returned, and, the uterus being stimulated to a vigorous action by the introduction of the hand, the head was thrown from the brim of the pelvis, and descended as in a natural presentation, the child being born in a short time. Hemorrhage again took place to a considerable extent at this stage; but on the administration of another dose of ergot, and the use of friction over the abdomen, uterine contractions came on, and the flooding ceased.

After waiting about fifteen minutes, one of the placentas was thrown off, to which were attached three of the umbilical cords; it was very large, having on its fœtal surface two distinct chambers. After waiting nearly an hour longer, I was under the necessity of introducing my hand and detaching the second one from near the fundus of the uterus; the attachment was slight. Tonic contraction of the uterus occurring, my patient was put to bed in safety, but, as might be expected, very much exhausted. She recovered rapidly, and, when I called to see the children, three weeks afterwards, was able to attend to her household duties. It may be proper to remark that the first child floated in its own waters, and likewise the last; the second and third floated together.

The following is the weight of each child, taken in the order of birth: 1st, a girl, 6 pounds; 2d, a girl, $4\frac{3}{4}$ pounds; 3d, a girl, $2\frac{1}{4}$ pounds; 4th, a boy, $6\frac{1}{2}$ pounds; making in all $19\frac{1}{2}$ pounds. The children were all well formed, and five weeks after birth were living and hearty. The mother is thirty-three years of age, and is the mother of ten children, including the last four. The father is a robust man, about forty years old.

*Postscript.*—Since writing the above, I have learned that the smallest child died about five weeks after its birth, probably from overfeeding.

Franklin, Illinois, November 5, 1847.

